# Pelvic floor dysfunction: prevalence and associated factors

**DOI:** 10.1186/s12889-023-16901-3

**Published:** 2023-10-14

**Authors:** Rocío Adriana Peinado-Molina, Antonio Hernández-Martínez, Sergio Martínez-Vázquez, Julián Rodríguez-Almagro, Juan Miguel Martínez-Galiano

**Affiliations:** 1 Department of Nursing, University of Jaen, Jaen, Spain; 2 Department of Nursing, Physiotherapy and Occupational Therapy, Ciudad Real, Faculty of Nursing, University of Castilla-La Mancha, Ciudad Real, Spain; 3 Consortium for Biomedical Research in Epidemiology and Public Health (CIBERESP), Madrid, Spain

**Keywords:** Pelvic floor dysfunction, Associated factors, Women´s health, Pelvic floor

## Abstract

**Background:**

Pelvic floor dysfunction in women encompasses a wide range of clinical disorders: urinary incontinence, pelvic organ prolapse, fecal incontinence, and pelvic-perineal region pain syndrome. A literature review did not identify any articles addressing the prevalence of all pelvic floor dysfunctions.

**Objective:**

Determine the prevalence of the group of pelvic floor disorders and the factors associated with the development of these disorders in women.

**Material and methods:**

This observational study was conducted with women during 2021 and 2022 in Spain. Sociodemographic and employment data, previous medical history and health status, lifestyle and habits, obstetric history, and health problems were collected through a self-developed questionnaire. The Pelvic Floor Distress Inventory (PFDI-20) was used to assess the presence and impact of pelvic floor disorders. Pearson's Chi-Square, Odds Ratio (OR) and adjusted Odds Ratio (aOR) with their respective 95% confidence intervals (CI) were calculated.

**Results:**

One thousand four hundred forty-six women participated. Urinary incontinence occurred in 55.8% (807) of the women, fecal incontinence in 10.4% (150), symptomatic uterine prolapse in 14.0% (203), and 18.7% (271) reported pain in the pelvic area. The following were identified as factors that increase the probability of urinary incontinence: menopausal status. For fecal incontinence: having had instrumental births. Factors for pelvic organ prolapse: number of vaginal births, one, two or more. Factors for pelvic pain: the existence of fetal macrosomia.

**Conclusions:**

The prevalence of pelvic floor dysfunction in women is high. Various sociodemographic factors such as age, having a gastrointestinal disease, having had vaginal births, and instrumental vaginal births are associated with a greater probability of having pelvic floor dysfunction. Health personnel must take these factors into account to prevent the appearance of these dysfunctions.

**Supplementary Information:**

The online version contains supplementary material available at 10.1186/s12889-023-16901-3.

## Key messages

What is already known on this topic

Pelvic floor dysfunction in women is a prevalent and underdiagnosed problem worldwide. A literature review did not identify any articles addressing the prevalence of all pelvic floor dysfunctions.

What this study adds

Data are provided that raises awareness about the magnitude of the problem and confirms and identifies factors that predispose women to have pelvic floor dysfunctions.

How this study might affect research, practice, or policy

Some factors or clinical practices may influence the incidence of certain pelvic floor disorders, and professionals must take these into account. Knowing these also helps to develop protocols and strategies that address the problem.

## Introduction

The pelvic floor comprises muscles, ligaments, and fascia; this integration is essential for the stability and muscle tone of the pelvic girdle, continence, urination/defecation, and sexuality, among others [1]. Pelvic floor problems have a complex and multifactorial pathophysiology that affects women's health and must be detected to offer the most appropriate care and treatment [2].

Pelvic floor dysfunction in women encompasses a wide range of clinical disorders: urinary incontinence, pelvic organ prolapse (descent of the uterus or other pelvic organs), fecal incontinence, and pelvic-perineal region pain syndrome [3–5]. It is a prevalent problem worldwide and underdiagnosed [4, 5]. The prevalence of pelvic floor problems worldwide ranges from 1.9% to 46.50% [6–8]. Thus, in healthy non-pregnant women, pelvic floor disorders are up to 25% [4, 9] in the US, and in Japan, 46.5% of adult women report at least one disorder [9, 10].

The impact and negative effects on women's health and quality of life are significant, with important consequences for physical and mental health [11–15]. Moreover, pelvic floor disorders are disabling and cause embarrassment to those who suffer from them, can lead to social isolation, affect the performance of tasks, cause loss of personal and intimate relationships, and reduce participation in leisure activities [8].

Most of the results obtained in other studies are not conclusive [11–14, 16], and authors recommend carrying out more research to lay the foundations for effective treatment, and effective individualized prevention focused on women to try to reduce the development of associated pathologies [17]. Furthermore, there are no studies providing recent information on the prevalence of all these pelvic floor disorders, highlighting the crucial significance of research on pelvic floor dysfunctions in Spain and in most of the countries in which studies on the subject have been carried out. Existing prevalence data, limited mainly to urinary incontinence, fails to encompass the entirety of pelvic floor disorders studied. However, this information is essential for developing updated prevention strategies and addressing the problem in a population with a longer life expectancy.

Given the above, this study aims to determine the presence of different pelvic floor dysfunctions in non-pregnant women and the factors associated with the development of these disorders.

## Materials and methods

### Design and subject selection

This observational study was conducted with women in 2021 and 2022 in Spain. The following exclusion criteria were established: women under 18 years of age, those who had difficulty understanding Spanish, were pregnant, had given birth within the previous 12 months, and those with mental health or cognitive disorders that could affect data collection.

It was necessary to recruit a sample size of 890 women based on the following criteria: a 95% confidence level, an absolute error precision of 3%, and a population prevalence of pelvic floor disorders of 25% [9]. The percentage of replacements needed was estimated as 10%. The women were recruited consecutively.

### Information sources and study variables

Participants were recruited extensively by publicly disseminating the research in centers where women engaged in various activities, women's associations, and neighborhood associations. Additionally, participants from social and educational groups and workshops at the health center were considered. Furthermore, information was spread among the nurse's patient roster through healthcare centers, encompassing elderly care facilities and day centers, among others. Data collection took place after women were recruited and informed consent was obtained. Subsequently, trained observers conducted interviews.

In addition, to assess the presence and impact of pelvic floor disorders, the Pelvic Floor Distress Inventory (PFDI-20) was used [18], which has been validated and used in a population similar to ours [18]. The PFDI-20 contemplates different perspectives and includes 20 items divided into 3 symptom scales: symptoms of pelvic organ prolapse (POPDI-6 subscale) (questions 1 to 6); colorectal-anal symptoms (CRADI-8 subscale) (questions 7–14); and urinary symptoms (UDI-6 subscale) (questions 15–20). The following key questions were used to determine the prevalence of the different dysfunctions. For the prevalence of prolapse, the criterion of symptomatic prolapse was used by means of an affirmative answer in item 3; for fecal incontinence, the sum of affirmative answers in items 9 and 10 was used as a criterion; for urinary incontinence, the sum of answers. affirmative responses to items 16 and 17, while for the prevalence of pelvic pain the affirmative response to item 20 was used.

Each question uses the following 0–4 response format, categorized into four levels of dysfunction: none, a little, moderate, or a lot. The minimum score for each subscale is 0 and the maximum is 100 points, referring to minimum and maximum dysfunction. The total score of the PFDI-20 is the sum of the three subscales, with a maximum score of 300.

### Patient and public involvement statement

None.

### Statistical analysis

First, descriptive statistics were carried out using absolute and relative frequencies, as well as means and standard deviation (SD) for quantitative variables and medians for ordinal responses. Next, the bivariate and multivariate analyses were performed between the presence of pelvic floor disorders and possible associated factors. For this purpose, the Pearson Chi-Square test was used, and the Odds Ratio (OR) and adjusted Odds Ratios (aOR) were estimated with their respective 95% confidence intervals (CI), using binary logistic regression in the latter. When the multivariate analysis was carried out, we opted for the backward step procedure to determine the main factors associated with pelvic floor problems. A statistically significant value was considered when the value of *p* ≤ 0.05.

The statistical program used for the analysis of the information has been SPSS 28.0.

## Results

One thousand four hundred forty six women participated. Their mean age was 44.3 years (SD = 14.68), and mean BMI was 25.0 (SD = 4.75). 85.7% (1239) women did not smoke, and 54.4% (786) drank occasionally.

Regarding personal and obstetric history, 28.9% (418) were postmenopausal, 33.0% (477) had some type of illness, and 78.2% (1131) had been pregnant. Regarding the type of birth, 67.2% (917) had had the experience of a vaginal birth, and 26.2% (379) an instrumental one (Table 1).

**Table 1:** Sociodemographic and clinical characteristics of the study sample (*N* = 1446)

Variable	n (%)	Mean (SD)
**Age**		44.3 (14.68)
< 30 years	220 (15.2)	
30–49.9 years	799 (55.3)	
50–69.9 years	349 (24.1)	
> = 70 years	78 (5.4)	
**BMI**		25.0 (4.75)
Normal weight < 25	830 (57.4)	
Overweight 25–29.9	405 (28.0)	
Obesity > = 30	211 (14.6)	
**Civil status**		
Single	334 (23.1)	
Separated	19 (1.3)	
Divorced	73 (5.1)	
Widowed	67 (4.6)	
Common-law couple	125 (8.6)	
Married	828 (57.3)	
**Education level**		
Primary level, uncompleted	69 (4.8)	
Primary level, completed	90 (6.2)	
Secondary level	97 (6.7)	
Baccalaureate	186 (12.9)	
University level	1004 (69.4)	
**Employment sector**		
Administration	126 (8.7)	
Agriculture/livestock	29 (2.0)	
Commerce	59 (4.1)	
Student	105 (7.3)	
Industry and construction	54 (3.7)	
Retired	165 (11.4)	
Self-employed	168 (11.6)	
Public servant	740 (51.2)	
**Income level**		
< 1000 euros	196 (13.6)	
1000–1999 euros	512 (35.4)	
2000–2999 euros	425 (29.4)	
> 3000 euros	313 (21.6)	
**Alcohol consumption**		
Never	351 (24.3)	
Occasionally	786 (54.3)	
Only weekends	144 (10.0)	
Frequently	142 (9.8)	
Daily	23 (1.6)	
**Smoking habit**		
No	1239 (85.7)	
Yes	207 (14.3)	
**Pregnancy**		
None	315 (21.8)	
One	194 (13.4)	
Two or more	937 (64.8)	
**Vaginal birth**		
None	475 (32.8)	
One	289 (20.0)	
Two or more	682 (47.2)	
**Instrumental birth**		
No	1067 (73.8)	
Yes	379 (26.2)	
**Tear**		
No	904 (62.5)	
Yes	542 (37.5)	
**Macrosomia (Missing = 2)**		
No	1250 (86.4)	
Yes	194 (13.4)	
**Menopause**		
No	1028 (71.1)	
Yes	418 (28.9)	
**Illness**		
No	969 (67.0)	
Yes	477 (33.0)	
**Cardiovascular disorder**		
No	1323 (91.5)	
Yes	123 (8.5)	
**Respiratory disorder**		
No	1408 (97.4)	
Yes	38 (2.6)	
**Endocrine disorder**		
No	1298 (89.8)	
Yes	148 (10.2)	
**Gynecological disorder**		
No	1404 (97.1)	
Yes	42 (2.9)	
**Musculoskeletal disorder**		
No	1353 (93.6)	
Yes	93 (6.4)	
**Neurological disorder**		
No	1412 (97.6)	
Yes	34 (2.4)	
**Neoplastic disease**		
No	1434 (99.2)	
Yes	12 (0.8)	
**Gastrointestinal disorder**		
No	1404 (97.1)	
Yes	42 (2.9)	
**Dermatological disorder**		
No	1424 (98.5)	
Yes	22 (1.5)	
**Mental health disorder**		
No	1421 (98.3)	
Yes	25 (1.7)	
**Nephro-urological disorder**		
No	1436 (99.3)	
Yes	10 (0.7)	
**Immunological disorder**		
No	1435 (99.2)	
Yes	11 (0.8)	
**Ophthalmology-ENT disorder**		
No	1421 (98.3)	
Yes	25 (1.7)	

*SD* standard deviation, *BMI* body mass index, *ENT* ear, nose, and throat

Urinary incontinence occurred in 55.8% (807) of the women, fecal incontinence in 10.4% (150), symptomatic uterine prolapse in 14.0% (203), and 18.7% (271) reported pain in the pelvic area. Table 2 presents the frequencies of responses to the questionnaire for intestinal, urinary, or pelvic symptoms (PFDI-20) (Table 2).

**Table 2:** Prevalence of self-reported pelvic floor dysfunction PFDI-20 questionnaire

Variable	No % (n)	Yes % (n)
**1.** **Do you usually experience pressure in the lower abdomen?**	76.3 (1104)	23.7 (342)
**2.** **Do you usually experience heaviness or dullness in the pelvic area?**	78.8 (1140)	21.2 (306)
**3.** **Do you usually have a bulge or something falling out that you can see or feel in the vaginal area?**	86.0 (1243)	14.0 (203)
**4.** **Do you usually have to push on the vagina or around the rectum to have a complete bowel movement?**	70.1 (1014)	29.9 (432)
**5.** **Do you usually experience a feeling of incomplete bladder emptying?**	64.0 (925)	36.0 (521)
**6. Do you ever have to push up in the vaginal area with your fingers to start or complete urination?**	97.0 (1402)	3.0 (44)
**7. Do you feel you need to strain too hard to have a bowel movement?**	64.0 (926)	36.0 (520)
**8. Do you feel you have not completely emptied your bowels at the end of a bowel movement?**	64.9 (938)	35.1 (508)
**9. Do you usually lose stool beyond your control if your stool is well formed?**	97.9 (1416)	2.1 (30)
**10. Do you usually lose stool beyond your control if you stool is loose or liquid?**	90.0 (1302)	10.0 (144)
**11. Do you usually lose gas from the rectum beyond your control?**	62.4 (902)	37.6 (544)
**12. Do you usually have pain when you pass your stool?**	84.0 (1214)	16.0 (232)
**13. Do you experience a strong sense of urgency and have to rush to the bathroom to have a bowel movement?**	80.3 (1161)	19.7 (285)
**14.** **Does part of your intestines ever pass through the rectum and bulge outside during or after a bowel movement?**	87.8 (1269)	12.2 (177)
**15.** **Do you usually experience frequent urination?**	45.5 (658)	54.5 (788)
**16.** **Do you usually experience urine leakage associated with a feeling of urgency; that is, a strong sensation of needing to go to the bathroom?**	73.2 (1059)	26.8 (387)
**17.** **Do you usually experience urine leakage related to laughing, coughing, or sneezing?**	55.6 (804)	44.4 (642)
**18. Do you usually experience small amounts of urine leakage (that is, drops)?**	65.7 (950)	34.3 (496)
**19.** **Do you usually experience difficulty emptying your bladder?**	85.5 (1237)	14.5 (209)
**20. Do you usually experience pain of discomfort in the lower abdomen or genital region?**	81.3 (1175)	18.7 (271)
**Pelvic function disorder results PFDI-20**		
Urinary incontinence	44.2 (639)	55.8 (807)
Fecal incontinence	89.6 (1296)	10.4 (150)
Prolapse	86.0 (1243)	14.0 (203)
Pelvic pain	81.3 (1175)	18.7 (271)

Regarding the intensity of the discomfort of each of these symptoms, the item that presented the highest median score of 3 points was "If you usually leak urine when you cough, sneeze or laugh" (Table 3).

**Table 3:** Impact of pelvic floor disorders

**Variable**	**How much does it bother you?**				**Median**
	**Not at all % (n) 1 point**	**A little % (n) 2 points**	**Moderately % (n) 3 points**	**A lot % (n) 4 points**	
**1. If you usually experience pressure in the lower abdomen**	30.8 (144)	43.4 (203)	20.3 (95)	5.6 (26)	2
**2. If you usually experience heaviness or dullness in the pelvic**	33.1 (146)	43.8 (193)	16.1 (71)	7.0 (31)	2
**3. If you usually have a bulge or something falling out that you can see or feel in the vaginal area**	57.2 (191)	24.9 (83)	9.9 (33)	8.1 (27)	1
**4. If you usually have to push on the vagina or around the rectum to have a complete bowel movement**	32.6 (175)	40.2 (216)	18.4 (99)	8.8 (47)	2
**5. Yes Do you usually experience a feeling of incomplete bladder emptying**	29.4 (181)	46.4 (286)	17.0 (105)	7.1 (44)	2
**6. If you ever have to push a “bulge” in the vaginal area with your fingers to start or complete urinating**	76.7 (145)	11.6 (22)	6.3 (12)	5.3 (10)	1
**7. If you feel you need to strain too hard to have a bowel movement**	20.5 (128)	40.0 (249)	26.2 (163)	13.3 (83)	2
**8. If you feel you have not completely emptied your bowels at the end of a bowel movement**	24.4 (144)	43.8 (259)	21.0 (124)	10.8 (64)	2
**9. If you usually lose stool beyond your control if your stool is well formed**	79.6 (133)	8.4 (14)	7.2 (12)	4.8 (8)	1
**10. If you usually lose stool beyond your control if you stool is loose or fluid**	49.4 (131)	12.5 (33)	12.5 (33)	25.7 (68)	2
**11. If you usually lose gas from the rectum beyond your control**	24.2 (153)	33.5 (212)	23.4 (148)	18.8 (119)	2
**12. If you usually have pain when you pass your stool**	33.0 (116)	24.4 (86)	28.4 (100)	14.2 (50)	2
**13. If you experience a strong sense of urgency and have to rush to the bathroom to have a bowel movement**	33.5 (131)	27.4 (107)	20.2 (79)	18.9 (74)	2
**14. If part of your intestines ever passes through the rectum and bulge outside during or after a bowel**	45.1 (130)	26.0 (75)	17.0 (49)	11.8 (34)	2
**15. If you usually experience frequent urination**	45.9 (380)	28.9 (239)	18.6 (154)	6.6 (55)	2
**16. If you usually experience urine leakage associated with a feeling of urgency; that is, a strong sensation of needing to go to the bathroom to urinate**	26.0 (128)	25.6 (126)	24.8 (122)	23.6 (116)	2
**17. If you usually experience urine leakage related to laughing, coughing, or sneezing**	17.3 (125)	30.0 (217)	23.9 (173)	28.8 (208)	3
**18. If you usually experience small amounts of urine leakage (that is, drops)**	22.0 (128)	30.0 (175)	21.8 (127)	26.2 (153)	2
**19. If you usually experience difficulty emptying your bladder**	39.0 (130)	21.3 (71)	20.7 (69)	18.9 (63)	2
**20. If you usually experience pain of discomfort in the lower abdomen or genital region**	28.6 (108)	31.6 (119)	27.1 (102)	12.7 (48)	2

**Fig. 1:**
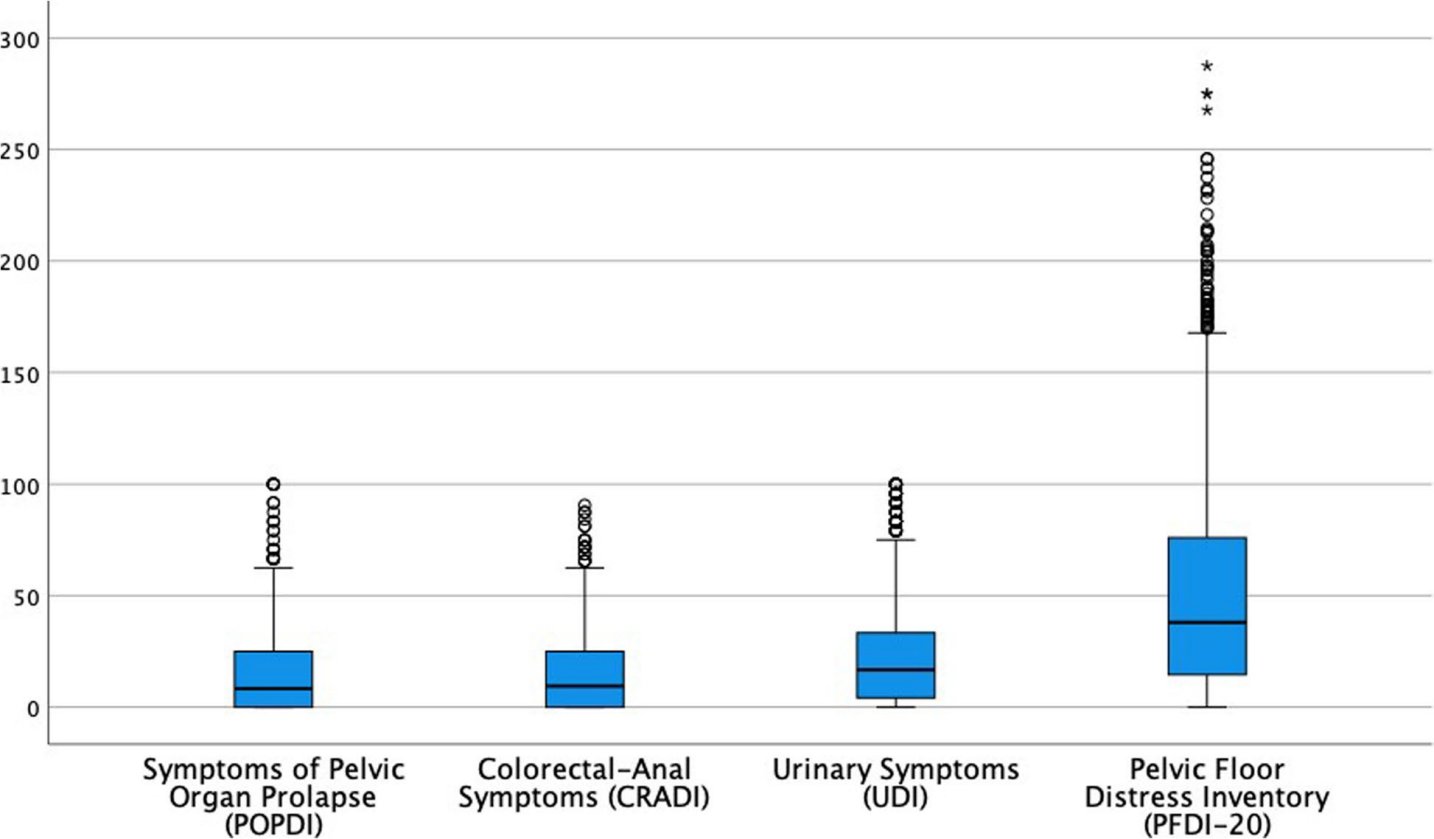
Distribution of PFD symptoms means (scores represented in PFDI-20 subscales)

The impact of pelvic organ prolapse symptoms had an average score of 14.33 points (SD = 16.63) on the POPDI-6 subscale, 15.69 points (SD = 16.90) on the CRADI-8 subscale for colorectal-anal symptoms, and an average score of 22.21 points (SD = 22.90) on the UDI-6 subscale for the impact of urinary symptoms. The PFDI-20 global scale presented an average of 52.23 points (SD = 49.0). Figure 1 shows the distribution of these scores.

Supplementary Table 1 also shows the bivariate analysis between the different sociodemographic variables, lifestyles, obstetric variables, and personal history and the different pelvic floor disorders. The variables that showed statistically significant associations with all pelvic floor disorders were age, BMI, menopause, number of pregnancies and vaginal births, instrumental birth, episiotomy, perineal laceration, and fetal macrosomia status (*p* < 0.05). Urinary incontinence and prolapse were also associated with a history of gastrointestinal pathology (*p* < 0.05). On the other hand, having a gastrointestinal and/or nephro-urological pathology was related to fecal incontinence. In addition, having a gynecological and/or gastrointestinal pathology showed a statistically significant association with pelvic pain (*p* < 0.05).

In Table 4, the following were identified as risk factors for urinary incontinence: older age (for each year the probability increases with aOR de 1.03; 95% CI: 1.02, 1.05), a high BMI (for each point of BMI the probability increases with aOR de 1.10; 95% CI: 1.07, 1.13), postmenopausal status compared to women pre-menopause increased their probability with a aOR de 1.89 (95% CI: 1.26, 2.82), number of births: women with one child increase their probability with respect to those who have not had any birth with a aOR de 2.61 (95% CI: 1.89, 3.60) women with two or more births increase their probability with respect to women without any births with aOR de 2.47 (95% CI: 1.86, 3.27) and have a gastrointestinal pathology with respect to those that do not have any problem, the probability increases with a aOR de 2.81(95% CI: 1.26, 6.29). Regarding fecal incontinence, factors that increase the probability were: oldest age (aOR: 1.03; 95% CI: 1.02, 1.04), highest BMI (for each point of BMI the risk increases with a aOR: 1.05; 95% CI: 1.01, 1.08), instrumental birth with respect to a normal delivery increases the risk with a aOR de 1.62; 95% CI: 1.12, 2.35) and have a gastrointestinal pathology (aOR: 3.19; 95% CI: 1.55, 6.55). Regarding factors that promote prolapse (Table 5), a relationship was observed with the number of vaginal births: one (aOR: 3.17; 95% CI: 1.84, 5.46) two or more (aOR: 3.10; 95% CI: 1.88, 5.04), instrumental birth (aOR: 1.80; 95% CI: 1.29, 2.50), fetal macrosomia with respect to normal weight, the probability increases with a aOR de 2.05 (95% CI: 1.40, 2.99), and gastrointestinal pathology (aOR: 2.45; 95% CI: 1.91, 5.02). A high BMI (aOR: 1.04; 95% CI: 1.02, 1.07), instrumental birth (aOR: 1.68; 95% CI: 1.26, 2.24), fetal macrosomia (aOR: 1.87; 95% CI: 1.31, 2.68), and have some type of gastrointestinal pathology (aOR: 2.07; 95% CI: 1.06, 4.07) were associated with a higher probability of having pelvic pain.

**Table 4:** Factors associated with pelvic floor problems. Multivariate analysis (Urinary incontinence and Fecal incontinence)

**Variable**	**Urinary incontinence**			**Fecal incontinence**		
	**No n (%)**	**Yes n (%)**	**aOR 95% CI:**	**No n (%)**	**Yes n (%)**	**aOR 95% CI:**
**Age (Mean SD)**	40.4 (13.50)	47.3 (14.87)	**1.03 (1.02, 1.05)**	43.4 (13.89)	52.1 (18.49)	**1.03 (1.02, 1.04)**
**BMI ( mean SD)**	23.8 (4.00)	25.9 (5.08)	**1.10 (1.07, 1.13)**	24.8 (4.65)	26.7 (5.30)	**1.05 (1.01, 1.08)**
**Menopause**						
No	492 (47.9)	536 (52.1)	1 (ref.)	950 (92.4)	78 (7.6)	
Yes	147 (35.2)	271 (64.8)	**1.89 (1.26, 2.82)**	346 (82.8)	72 (17.2)	
**Number of pregnancies**				357 (73.5)		
None	208 (66.0)	107 (34.0)		292 (92.7)	23 (7.3)	
One	95 (49.0)	99 (51.0)		173 (89.2)	21 (10.8)	
Two or more	336 (35.9)	601 (64.1)		831 (88.7)	106 (11.3)	
**Number of vaginal births**						
None	301 (63.4)	174 (36.6)	1 (ref.)	444 (93.5)	31 (6.5)	
One	104 (36.0)	185 (64.0)	**2.61 (1.89, 3.60)**	257 (88.9)	32 (11.1)	
Two or more	234 (34.3)	448 (65.7)	**2.47 (1.86, 3.27)**	595 (87.2)	87 (12.8)	
**Instrumental birth**						
No	517 (48.5)	550 (51.5)		978 (91.7)	89 (8.3)	1 (ref.)
Yes	122 (32.2)	257 (67.8)		318 (83.9)	61 (16.1)	**1.62 (1.12, 2.35)**
**Episiotomy**						
No	415 (54.6)	345 (45.4)		700 (92.1)	60 (7.9)	
Yes	224 (32.7)	462 (67.3)		596 (86.9)	90 (13.1)	
**Perineal tear**						
No	455 (50.3)	449 (49.7)		827 (91.5)	77 (8.5)	
Yes	184 (33.9)	358 (66.1)		469 (86.5)	73 (13.5)	
**Fetal macrosomia**						
No	579 (46.3)	671 (53.7)		1131 (90.5)	119 (9.5)	
Yes	60 (30.9)	134 (69.1)		163 (84.0)	31 (16.0)	
**Smoker**						
No	540 (43.6)	699 (56.4)		1114 (89.9)	125 (10.1)	
Yes	99 (47.8)	108 (52.2)		182 (87.9)	25 (12.1)	
**Gynecological pathology**						
No	622 (44.3)	782 (55.7)		1259 (89.7)	145 (10.3)	
Yes	17 (40.5)	25 (59.5)		37 (88.1)	5 (11.9)	
**Gastrointestinal pathology**						
No	630 (44.9)	774 (55.1)	1 (ref.)	1267 (90.2)	137 (9.8)	1 (ref.)
Yes	9 (21.4)	33 (78.6)	**2.81 (1.26, 6.29)**	29 (69.0)	13 (31.0)	**3.19 (1.55, 6.55)**
**Nephro-urological pathology**						
No	637 (44.4)	799 (55.6)		1290 (89.8)	146 (10.2)	
Yes	2 (20.0)	8 (80.0)				

*aOR* adjusted odds ratio, *BMI *body mass index, *CI *confidence interval, *OR *odds ratio, *ref*. reference

**Table 5:** Factors associated with pelvic floor problems. Multivariate analysis (Prolapse and Pain)

**Variable**	**Prolapse**			**Pain**		
	**No n (%)**	**Yes n (%)**	**aOR 95% CI:**	**No n (%)**	**Yes n (%)**	**aOR 95% CI:**
**Age (Mean SD)**	43.5 (14.36)	48.9 (15.74)		43.7 (14.44)	46.7 (15.44)	
**BMI ( mean SD)**	24.9 (4.73)	25.5 (4.84)		24.75 (4.62)	26.1 (5.14)	**1.04 (1.02, 1.07)**
**Menopause**						
No	899 (87.5)	129 (12.5)		854 (83.1)	174 (16.9)	
Yes	344 (82.3)	74 (17.7)		321 (76.8)	97 (23.2)	
**Number of pregnancies**						
None	302 (95.2)	13 (4.1)		272 (86.3)	43 (13.7)	
One	165 (85.1)	29 (14.9)		163 (84.0)	31 (16.0)	
Two or more	776 (82.8)	161 (17.2)		740 (79.0)	197 (21.0)	
**Number of vaginal births**						
None	452 (95.2)	23 (4.8)	1 (ref.)	404 (85.1)	71 (14.9)	
One	239 (82.7)	50 (17.3)	**3.17 (1.84, 5.46)**	224 (77.5)	65 (22.5)	
Two or more	552 (80.9)	130 (19.1)	**3.10 (1.88, 5.04)**	547 (80.2)	135 (19.8)	
**Instrumental birth**						
No	957 (89.7)	110 (10.3)	1 (ref.)	896 (84.0)	171 (16.0)	1 (ref.)
Yes	286 (75.5)	93 (24.5)	**1.80 (1.29, 2.50)**	279 (73.6)	100 (26.4)	**1.68 (1.26, 2.24)**
**Episiotomy**						
No	687 (90.4)	73 (9.6)		639 (84.1)	121 (15.9)	
Yes	556 (81.0)	130 (19.0)		536 (78.1)	150 (21.9)	
**Perineal tear**						
No	805 (89.0)	99 (11.0)		757 (83.7)	147 (16.3)	
Yes	438 (80.8)	104 (19.2)		418 (77.1)	124 (22.9)	
**Fetal macrosomia**						
No	1101 (88.1)	149 (11.9)	1 (ref.)	1042 (83.4)	208 (16.6)	1 (ref.)
Yes	141 (72.7)	53 (27.3)	**2.05 (1.40, 2.99)**	132 (68.0)	62 (32.0)	**1.87 (1.31, 2.68)**
**Smoker**						
No	1059 (85.5)	180 (14.5)		996 (80.4)	243 (19.6)	
Yes	184 (88.9)	23 (11.1)		179 (86.5)	28 (13.5)	
**Gynecological pathology**						
No	1208 (86.0)	196 (14.0)		1146 (81.6)	258 (18.4)	
Yes	35 (83.3)	7 (16.7)		29 (69.0)	13 (31.0)	
**Gastrointestinal pathology**						
No	1213 (86.4)	191 (13.6)	1 (ref.)	1147 (81.7)	257 (18.3)	1 (ref.)
Yes	30 (71.4)	12 (28.6)	**2.45 (1.91, 5.02)**	28 (66.7)	14 (33.3)	**2.07 (1.06, 4.07)**
**Nephro-urological pathology**						
No	1234 (85.9)	202 (14.1)		1169 (81.4)	267 (18.6)	
Yes	9 (90.0)	1 (10.0)		6 (60.0)	4 (40.0)	

## Discussion

The presence in women of pelvic floor dysfunctions is high. More than half of women have some type of urinary incontinence, one in ten reports having fecal incontinence to some degree, a similar number report having symptoms of pelvic organ prolapse, and approximately one fifth of women report pelvic pain. Age, BMI, menopausal status, the number of vaginal births, having instrumental births, fetal macrosomia, and gastrointestinal pathology were identified as being associated with pelvic floor dysfunction.

We attempted to control for confounding bias by using multivariate analysis, including in the model all those variables that could influence the results, and by the selection of the participating women. Although the presence of memory bias and selection bias has been considered, we do not believe that they are present in the end as they have been controlled with the adaptation of the language, making it easy to read and understand at all educational levels. Although clinical evaluation of the women was not carried out using clinical diagnostic means, the questionnaires used, which have been validated and used in a population with similar characteristics to ours, are internationally accepted as detection methods for pelvic floor disorders; therefore, we do not consider that it could have affected the results obtained [19, 20].

In countries similar to Spain, various cross-sectional descriptive studies [21, 22], where women with a similar age range to our study participated, report a prevalence of urinary incontinence higher than that found in our results, reaching a difference of up to 30 percent. However, these studies were carried out in very defined and specific places, whereas our study was carried out with women from all over the country. Likewise, worldwide, our results report a higher prevalence of urinary incontinence than those obtained in various other countries, such as the USA [23] in a nationally representative population of women, and in Qatar [24], the United Arab Emirates [25], and Oman [26]. However, the results found in a study carried out in the United Kingdom, reported a presence of urinary incontinence 10 percentage points higher than what we observed [27].

A descriptive observational study [9] carried out in the USA with 1961 women showed a rate of 2.9% for prolapse, results lower than those obtained in the present study, which found 14%. In this same country, USA, another study related to prolapse published by the American College of Obstetricians and Gynecologists [28] found that 3–6% of women reported symptoms related to prolapse; however, after gynecological examination of the women, the prevalence of prolapse (non-symptomatic) was 41%-50%, despite having prolapse less than 10% of them perceived and manifested symptoms. This may lead one to think that the prevalence of prolapse without symptoms in our sample may be much higher; however, we cannot confirm this.

In a systematic review [29] that included 38 studies, in which the majority of women participated, they found a median rate of fecal incontinence of 8.9%. Within this review, a very wide range of variability was observed, with prevalence ranging from 2.0% to 20.7%. In the USA, the prevalence stood at 9.4%, which is slightly lower but close to those obtained in the present study, 10.4%.

Regarding the rate of pelvic pain, our research reported 18.7%, in line with what Ahangari [30] reported in a systematic review that only found 7 articles with different types of studies (cross-sectional, community study, etc.) that addressed the issue of chronic pelvic pain.

Regarding urinary incontinence, our results coincide with those of Al-Badr et al. [5] in a cross-sectional study with 2289 women using the same method and identification instruments of pelvic floor disorders as in our study, which revealed in a bivariate analysis that age, parity, instrumental births, vaginal births and menopause were also associated with these disorders. However, statistical significance was not maintained in some factors after multivariable analysis. Contrary to our results, there is no agreement that vaginal births and the menopausal state are associated with this dysfunction. Hage-Fransen et al. [31] conducted a systematic review and meta-analysis that included 40 articles on urinary incontinence and concluded that this dysfunction is not associated with the type of vaginal birth. On the other hand, the descriptive observational study carried out by Luna et al. [32] in Japan (1222 randomly selected women in 15 hospitals) coincided with our results, identifying menopause as a predictor of urinary incontinence.

Regarding fecal incontinence, De Souza [33] in his cross-sectional descriptive study carried out in Brazil on 342 women, found that age and an increase in BMI are related to this dysfunction, as other authors have also previously cited [31]. In addition, this author identified that gastrointestinal pathologies, either caused by a surgical history or naturally, were associated with this dysfunction; all of these factors mentioned (age, BMI, and gastrointestinal pathologies) are in line with our results. Likewise, in the case–control study including 68 women in the United States conducted by Bharucha et al. [34], gastrointestinal pathology was also identified as a factor associated with the presence of pelvic floor disorders.

Al-Badr et al. [5], De Souza [33] and Hage-Fransen et al. [31] found associations between instrumental birth and the probability of develop fecal incontinence in accordance with our results. Also in line with our results [35], Hage-Fransen et al. [31] reported an association between the presence of fetal macrosomia (newborn > 4000 g) and a greater presence of this disorder.

Regarding uterine prolapse, Swift et al. [36] in a multicenter observational study carried out with 1004 women between 18 and 83 years old, identified an association between this disorder and a high BMI, results that do not coincide with those obtained in our study. This association, however, was also found by other authors such as Kim et al. [37]. On the other hand, Swift et al. [36] found, in line with what was detected in our results, that fetal macrosomia was associated with uterine prolapse. The results obtained in a systematic review and meta-analysis [38], which included 9 articles on pelvic organ prolapse, conclude that high parity and instrumental birth are associated with this disorder, in accordance with our results.

Díaz-Mohedo et al. [39] studied the factors that were associated with the presence of pelvic pain. They conducted a study with 887 people, of whom 414 were women, aged 18 to 65 years, finding that 30.9% of women experienced pelvic pain, a higher rate than our study. Moreover, in contrast to our study, these authors state that they found no association with BMI, type of birth, or fetal macrosomia.

Urinary incontinence and fecal incontinence increase significantly with increasing age. Likewise, an increase in BMI was associated with a greater probability of developing urinary incontinence, fecal incontinence, and pelvic pain. It is important to highlight this factor as it can be modifiable with appropriate interventions [9, 31, 32]. Likewise, some clinical practices such as instrumental births may influence the incidence of certain pelvic floor disorders, so healthcare professionals must be aware of this and take into account the short, medium, and long-term consequences that instrumental births can have. The results highlight the impact and importance of this public health problem, urging more research on pelvic floor disorders. The need for specific consultations, training, treatment, and prevention to avoid pelvic floor disorders is a priority for various authors. Specifically, knowledge of the prevalence of pelvic floor disorders and factors associated with it can enhance the development of targeted prevention strategies, including personalized exercise regimens, pre- and postnatal care, and lifestyle modifications, aimed at reducing the incidence and severity of pelvic floor disorders. It is necessary to implement measures that mitigate the associated factors and carry out research that addresses how these measures help reduce the presence of pelvic floor dysfunctions.

## Conclusions

The prevalence of pelvic floor disorders is high, and around 40% of women present a single problem, around 17% have two disorders, approximately 6% have three problems, and around 2% have four. Urinary incontinence is the most frequent pelvic floor problem, followed by pelvic pain, symptoms of pelvic organ prolapse, and fecal incontinence.

Gastrointestinal pathology is associated with all the pelvic floor disorders studied. Age was associated with a greater presence of urinary incontinence and fecal incontinence. A high BMI was identified with a greater possibility of urinary and fecal incontinence and pelvic pain. Menopause has been linked to an increased likelihood of urinary incontinence. Instrumental birth was associated with higher rates of fecal incontinence, uterine prolapse, and pelvic pain. Fetal macrosomia was also associated with uterine prolapse and pelvic pain. Parity is associated with the presence of urinary incontinence and prolapse.

### Supplementary Information

**Additional file 1:** **Supplementary Table 1.** Factors associated with pelvic floor problems. Bivariate analysis.

## Data Availability

The data that support the findings of this study are available from the corresponding author upon reasonable request.
