# Supplementary material for: Pelvic floor dysfunction: prevalence and associated factors
Source: BMC Public Health. 2023 Oct 14;23:2005. doi: 10.1186/s12889-023-16901-3 (PMC10576367; doi:10.1186/s12889-023-16901-3)
Supplement: Supplementary file 1 — Additional file 1: Supplementary Table 1. Factors associated with pelvic floor problems. Bivariate analysis. [file 12889_2023_16901_MOESM1_ESM.docx]

1. **Supplementary Material**

**Supplementary Table 1. Factors associated with pelvic floor problems. Bivariate analysis**

|  | | **Urinary incontinence** | | | | | **Fecal incontinence** | | | **Prolapse** | | | **Pain** | | |
| --- | --- | --- | --- | --- | --- | --- | --- | --- | --- | --- | --- | --- | --- | --- | --- |
| **Variable** | | **No**  **n (%)** | | **Yes**  **n (%)** | **OR 95% CI:** | | **No**  **n (%)** | **Yes**  **n (%)** | **OR 95% CI:** | **No**  **n (%)** | **Yes**  **n (%)** | **OR 95% CI:** | **No**  **n (%)** | **Yes**  **n (%)** | **OR 95% CI:** |
| **Age (Mean SD)** | | 40.4 (13.50) | | 47.3 (14.87) | 1 (ref.)  1.04 (1.03–1.04) | | 43.4 (13.89) | 52.1 (18.49) | 1 (ref.)  1.04 (1.03–1.05) | 43.5 (14.36) | 48.9 (15.74) | 1 (ref.)  1.02 (1.01–1.03) | 43.7 (14.44) | 46.7 (15.44) | 1 (ref.)  1.01 (1.01–1.02) |
| **BMI ( mean SD)** | | 23.8 (3.4) | | 25.6 (5.08) | 1 (ref.)  1.11 (1.09–1.14) | | 24.8 (4.65) | 26.7 (5.30) | 1 (ref.)  1.07 (1.04–1.11) | 24.9 (4.73) | 25.5 (4.84) | 1 (ref.)  1.02 (0.99–1.05) | 24.75 (4.62) | 26.1 (5.14) | 1 (ref.)  1.06 (1.03–1.09) |
| **Menopause** | |  | |  |  | |  |  |  |  |  |  |  |  |  |
| **No** | | 492 (47.9) | | 536 (52.1) | 1 (ref.) | | 950 (92.4) | 78 (7.6) | 1 (ref.) | 899 (87.5) | 129 (12.5) | 1 (ref.) | 854 (83.1) | 174 (16.9) | 1 (ref.) |
| Yes | | 147 (35.2) | | 271 (64.8) | 1.69 (1.34, 2.14) | | 346 (82.8) | 72 (17.2) | 2.53 (1.80, 3.57) | 344 (82.3) | 74 (17.7) | 1.50 (1.10, 2.05) | 321 (76.8) | 97 (23.2) | 1.48 (1.12, 1.96) |
| **Number of pregnancies** | |  | |  |  | | 357 (73.5) |  |  |  |  |  |  |  |  |
| None | | 208 (66.0) | | 107 (34.0) | 1 (ref.) | | 292 (92.7) | 23 (7.3) | 1 (ref.) | 302 (95.2) | 13 (4.1) | 1 (ref.) | 272 (86.3) | 43 (13.7) | 1 (ref.) |
| One | | 95 (49.0) | | 99 (51.0) | 2. 03 (1.41, 2.92) | | 173 (89.2) | 21 (10.8) | 1.54 (0.83, 2.87) | 165 (85.1) | 29 (14.9) | 4.08 (2.07, 8.07) | 163 (84.0) | 31 (16.0) | 1.20 (0.73, 1.99) |
| Two or more | | 336 (35.9) | | 601 (64.1) | 3.48 (2.66, 4.55) | | 831 (88.7) | 106 (11.3) | 1.62 (1.01, 2.60) | 776 (82.8) | 161 (17.2) | 4.82 (2.70, 8.61) | 740 (79.0) | 197 (21.0) | 1.68 (1.18, 2.41) |
| **Number of vaginal births** | |  | |  |  | |  |  |  |  |  |  |  |  |  |
| None | | 301 (63.4) | | 174 (36.6) | 1 (ref.) | | 444 (93.5) | 31 (6.5) | 1 (ref.) | 452 (95.2) | 23 (4.8) | 1 (ref.) | 404 (85.1) | 71 (14.9) | 1 (ref.) |
| One | | 104 (36.0) | | 185 (64.0) | 3.08 (2.27, 4.17) | | 257 (88.9) | 32 (11.1) | 1.78 (1.06, 2.99) | 239 (82.7) | 50 (17.3) | 4.11 (2.45, 6.90) | 224 (77.5) | 65 (22.5) | 1.65 (1.14, 2.40) |
| Two or more | | 234 (34.3) | | 448 (65.7) | 3.31 (2.59, 4.23) | | 595 (87.2) | 87 (12.8) | 2.09 (1.37, 3.21) | 552 (80.9) | 130 (19.1) | 4.63 (2.92, 7.34) | 547 (80.2) | 135 (19.8) | 1.40 (1.03, 1.92) |
| **Instrumental birth** | |  | |  |  | |  |  |  |  |  |  |  |  |  |
| No | | 517 (48.5) | | 550 (51.5) | 1 (ref.) | | 978 (91.7) | 89 (8.3) | 1 (ref.) | 957 (89.7) | 110 (10.3) | 1 (ref.) | 896 (84.0) | 171 (16.0) | 1 (ref.) |
| Yes | | 122 (32.2) | | 257 (67.8) | 1.98 (1.55, 2.53) | | 318 (83.9) | 61 (16.1) | 2.11 (1.49, 2.99) | 286 (75.5) | 93 (24.5) | 2.83 (2.08, 3.84) | 279 (73.6) | 100 (26.4) | 1.88 (1.42, 2.49) |
| **Episiotomy** | |  | |  |  | |  |  |  |  |  |  |  |  |  |
| No | | 415 (54.6) | | 345 (45.4) | 1 (ref.) | | 700 (92.1) | 60 (7.9) | 1 (ref.) | 687 (90.4) | 73 (9.6) | 1 (ref.) | 639 (84.1) | 121 (15.9) | 1 (ref.) |
| Yes | | 224 (32.7) | | 462 (67.3) | 2.48 (2.00, 3.07) | | 596 (86.9) | 90 (13.1) | 1.76 (1.25, 2.49) | 556 (81.0) | 130 (19.0) | 2.20 (1.62, 2.99) | 536 (78.1) | 150 (21.9) | 1.48 (1.13, 1.93) |
| **Perineal tear** | |  | |  |  | |  |  |  |  |  |  |  |  |  |
| No | | 455 (50.3) | | 449 (49.7) | 1 (ref.) | | 827 (91.5) | 77 (8.5) | 1 (ref.) | 805 (89.0) | 99 (11.0) | 1 (ref.) | 757 (83.7) | 147 (16.3) | 1 (ref.) |
| Yes | | 184 (33.9) | | 358 (66.1) | 1.97 (1.58, 2.46) | | 469 (86.5) | 73 (13.5) | 1.67 (1.19, 2.35) | 438 (80.8) | 104 (19.2) | 1.93(1.43, 2.60) | 418 (77.1) | 124 (22.9) | 1.53 (1.17, 2.00) |
| **Fetal macrosomia** | |  | |  |  | |  |  |  |  |  |  |  |  |  |
| No | | 579 (46.3) | | 671 (53.7) | 1 (ref.) | | 1131 (90.5) | 119 (9.5) | 1 (ref.) | 1101 (88.1) | 149 (11.9) | 1 (ref.) | 1042 (83.4) | 208 (16.6) | 1 (ref.) |
| Yes | | 60 (30.9) | | 134 (69.1) | 1.93 (1.40, 2.27) | | 163 (84.0) | 31 (16.0) | 1.80 (1.18, 2.77) | 141 (72.7) | 53 (27.3) | 2.78 (1.94, 3.98) | 132 (68.0) | 62 (32.0) | 2.35 (1.68, 3.30) |
| **Smoker** | |  | |  |  | |  |  |  |  |  |  |  |  |  |
| No | | 540 (43.6) | | 699 (56.4) | 1 (ref.) | | 1114 (89.9) | 125 (10.1) | 1 (ref.) | 1059 (85.5) | 180 (14.5) | 1 (ref.) | 996 (80.4) | 243 (19.6) | 1 (ref.) |
| Yes | | 99 (47.8) | | 108 (52.2) | 0.84 (0.63, 1.13) | | 182 (87.9) | 25 (12.1) | 1.22 (0.78, 1.93) | 184 (88.9) | 23 (11.1) | 0.74 (0.46, 1.17) | 179 (86.5) | 28 (13.5) | 0.64 (0.42, 0.98) |
| **Gynecological pathology** | |  | |  |  | |  |  |  |  |  |  |  |  |  |
| No | | 622 (44.3) | | 782 (55.7) | 1 (ref.) | | 1259 (89.7) | 145 (10.3) | 1 (ref.) | 1208 (86.0) | 196 (14.0) | 1 (ref.) | 1146 (81.6) | 258 (18.4) | 1 (ref.) |
| Yes | | 17 (40.5) | | 25 (59.5) | 1.17 (0.62, 2.19) | | 37 (88.1) | 5 (11.9) | 1.17 (0.45, 3.03) | 35 (83.3) | 7 (16.7) | 1.23 (0.54, 2.81) | 29 (69.0) | 13 (31.0) | 1.99 (1.02, 3.88) |
| **Gastrointestinal pathology** | |  | |  |  | |  |  |  |  |  |  |  |  |  |
| No | | 630 (44.9) | | 774 (55.1) | 1 (ref.) | | 1267 (90.2) | 137 (9.8) | 1 (ref.) | 1213 (86.4) | 191 (13.6) | 1 (ref.) | 1147 (81.7) | 257 (18.3) | 1 (ref.) |
| Yes | | 9 (21.4) | | 33 (78.6) | 2.98 (1.42, 6.28) | | 29 (69.0) | 13 (31.0) | 4.15 (2.10, 8.16) | 30 (71.4) | 12 (28.6) | 2.54 (1.28, 5.05) | 28 (66.7) | 14 (33.3) | 2.23 (1.16, 4.30) |
| **Nephro-urological pathology** | |  | |  |  | |  |  |  |  |  |  |  |  |  |
| No | | 637 (44.4) | | 799 (55.6) | 1 (ref.) | | 1290 (89.8) | 146 (10.2) | 1 (ref.) | 1234 (85.9) | 202 (14.1) | 1 (ref.) | 1169 (81.4) | 267 (18.6) | 1 (ref.) |
| Yes | | 2 (20.0) | | 8 (80.0) | 3.19 (0.68, 15.07) | | 6 (60.0) | 4 (40.0) | 5.89 (1.64, 21.12) | 9 (90.0) | 1 (10.0) | 0.68 (0.09, 5.39) | 6 (60.0) | 4 (40.0) | 2.92 (0.82, 10.42) |
|  |  | |  | |  |  | | | | | | | | | |
